# Estimating the force of infection of four dengue serotypes from serological studies in two regions of Vietnam

**DOI:** 10.1371/journal.pntd.0012568

**Published:** 2024-10-07

**Authors:** Huynh Thi Phuong, Nguyen Ha Thao Vy, Nguyen Thi Le Thanh, Maxine Tan, Erwin de Bruin, Marion Koopmans, Maciej F. Boni, Hannah E. Clapham

**Affiliations:** 1 Oxford University Clinical Research Unit, Wellcome Trust Major Overseas Programme, Ho Chi Minh City, Vietnam; 2 Institute of Epidemiology and Social Medicine, University of Münster, Münster, Germany; 3 Saw Swee Hock School of Public Health, National University of Singapore and National University Health System, Singapore, Singapore; 4 Nuffield Department of Medicine, University of Oxford, Oxford, United Kingdom; 5 Department of Viroscience, Erasmus MC, Rotterdam, Netherlands; 6 Center for Infectious Disease Dynamics, Department of Biology, Pennsylvania State University, University Park, Pennsylvania, Unites States of America; NIAID Integrated Research Facility, UNITED STATES OF AMERICA

## Abstract

Dengue is endemic in Vietnam with circulation of all four serotypes (DENV1-4) all year-round. It is hard to estimate the disease’s true serotype-specific transmission patterns from cases due to its high asymptomatic rate, low reporting rate and complex immunity and transmission dynamics. Seroprevalence studies have been used to great effect for understanding patterns of dengue transmission. We tested 991 population serum samples (ages 1–30 years, collected 2013 to 2017), 531 from Ho Chi Minh City and 460 from Khanh Hoa in Vietnam, using a flavivirus protein microarray assay. By applying our previously developed inference framework to the antibody profiles from this assay, we can (1) determine proportions of a population that have not been infected or infected, once, or more than once, and (2) infer the infecting serotype in those infected once. With these data, we then use mathematical models to estimate the force of infection (FOI) for all four DENV serotypes in HCMC and KH over 35 years up to 2017. Models with time-varying or serotype-specific DENV FOI assumptions fit the data better than constant FOI. Annual dengue FOI ranged from 0.005 (95%CI: 0.003–0.008) to 0.201 (95%CI: 0.174–0.228). FOI varied across serotypes, higher for DENV1 (95%CI: 0.033–0.048) and DENV2 (95%CI: 0.018–0.039) than DENV3 (95%CI: 0.007–0.010) and DENV4 (95%CI: 0.010–0.016). The use of the PMA on serial age-stratified cross-sectional samples increases the amount of information on transmission and population immunity, and should be considered for future dengue serological surveys, particularly to understand population immunity given vaccines with differential efficacy against serotypes, however, there remains limits to what can be inferred even using this assay.

## Author summary

Dengue is endemic in Vietnam and this virus consists of four serotypes (DENV1-4) that circulate- all year-round. It has complex transmission and immunity dynamics, which makes serotype-specific transmission patterns difficult to estimate. Moreover, high asymptomatic rates which leads to under reporting and little serotype testing of cases adds to this issue. Many studies have shown that seroprevalence studies are useful to understanding dengue transmission patterns, hence, we undertook a seroprevalence studies in Ho Chi Minh City and Khanh Hoa, Vietnam. We tested samples using a flavivirus protein microarray (PMA) serological assay and used previously developed frameworks to estimate past infection exposures for each individual. With these past exposure histories, we estimated the transmission intensity for all four dengue serotypes. We found this method provided substantial information on past serotype transmission. We estimated the annual dengue force of infection (FOI) ranged from 0.005 (95%CI: 0.003–0.008) to 0.201 (95%CI: 0.174–0.228). FOI varied across serotypes, higher for DENV1 (95%CI: 0.033–0.048) and DENV2 (95%CI: 0.018–0.039) than DENV3 (95%CI: 0.007–0.010) and DENV4 (95%CI: 0.010–0.016). This method can be used for planning future control measures, especially vaccination.

## Introduction

Dengue is a vector-borne disease that results from infection by one of the four dengue virus serotypes (DENV1-4) [1]⁠. Two species of mosquitoes, *Aedes aegypti* and *Aedes albopictus* are known to transmit the viral pathogen [1]. Dengue is endemic in most countries in Africa, the Americas, the Eastern Mediterranean, South-East Asia, and the Western Pacific Region [1]. It is estimated that up to approximately 294 million (95% CI: 217–392) infections result in either no discernible symptoms or mild symptoms each year, such that these cases go undetected by public health surveillance systems (inapparent), while 96 million (95% CI: 67–136) infected individuals manifest clinically (apparent) [2]. It is estimated that in 2013, dengue was accountable for 1.14 million Disability-Adjusted Life Years (DALYs) when both fatal and non-fatal outcomes were considered [3].

DENV causes a wide range of manifestations in humans, from mild to life-threatening severe dengue, previously called Dengue Haemorrhagic Fever (DHF) or Dengue Shock Syndrome (DSS) [4]. A person’s first dengue infection is most likely to result in minor or no symptoms, conferring lifelong immunity to the infecting serotype [5]^⁠^. However, secondary infection with another serotype has been reported to be more likely cause severe disease [6–9]. There are no specific antiviral drugs or treatments for severe dengue [10]. There are a number of vaccines at different stages of development, licensure and approval, and the use of vaccines could be an effective measure to stop the spreading and thus reduce the burden of the disease [11].

Vietnam is a dengue-endemic setting; despite the country’s effort to significantly reduce the scale, frequency, and impact of dengue outbreaks, dengue burden is still substantial [12]⁠. The number of reported cases varies each year, in 2019, Vietnam recorded more than 320,000 dengue cases [13], though this and other similarly reported case counts are based on hospitalization data only. All four DENV serotypes have circulated in Vietnam [14].

In the study of DENV epidemiology, complete reliance on only case-based surveillance methods to determine disease transmission dynamics will result in the under-reporting of disease in the population due to the virus’s wide range of manifestations and often the serotype is not reported. It has been found that only a proportion of infected individuals experience symptoms (apparent), with estimates ranging from approximately 13% to 50% [2,15]. Only a small proportion of these individuals (3–13%) will experience symptoms severe enough to require hospitalisation [4,16–18]. The different serotypes may also vary in their propensity to cause disease depending on an individual’s immune status, such as DENV2 and DENV4 being more likely to cause disease in the presence of dengue antibody than without the dengue antibody [19]. As a result, inferring the full picture of past transmission from case data alone is difficult. Hence, serological studies can be used to better understand the full picture of past DENV transmission [20].

Currently, due to their ease of use, enzyme-linked immunosorbent assays (IgG ELISAs) are among the most common methods used, despite some commercial kits being vastly expensive. However, these approaches are unable to differentiate specific dengue serotypes and are influenced by cross-reactivity with other flaviviruses. The plaque reduction neutralisation test (PRNT) provides better information with serotype specificity and discrimination between single and multiple past exposures, but it is expensive and more labour intensive. In contrast, renowned for its high-throughput capabilities, the flavivirus protein microarray (PMA) method provides a cost-effective alternative. Especially, it can distinguish between dengue serotypes thanks to the highly immunogenic recombinant protein NS1 used for expressing specific dengue viruses [21]⁠. This unique ability positions the PMA method as a promising tool for deployment in sero-epidemiological studies within dengue-endemic settings.

Sero-epidemiological studies aid in bridging the gap between apparent and inapparent dengue cases, providing a comprehensive depiction of the current landscape of population immunity, from which we can infer complete past transmission dynamics. The use of age-stratified serological surveys in the study of DENV epidemiology has been instrumental in gaining a better understanding of transmission dynamics and intensity [22–24]⁠, allowing for the detection of outbreaks and the study of infection trends. Policymakers, equipped with this knowledge of the current infection landscape, can make informed decisions regarding the implementation of control interventions, such as the introduction of DENV vaccines or enhance vector control in high-risk regions. In this study, we used a novel serological assay [21]⁠ on general-population samples collected for a serum bank in central and southern Vietnam. Combining the data with transmission models [25], we estimate the transmission intensity by location, serotype, and over time in two locations.

## Materials and methods

### Ethics statement

The Scientific and Ethical Committee of the Hospital for Tropical Diseases in Ho Chi Minh City and the Oxford Tropical Research Ethics Committee at the University of Oxford approved the study. The samples were anonymized residual samples from routine tests, in this approval consent was not sought from each individual [26].

### Study design

#### The population samples

As described previously [27,28]⁠, serum samples used in this study were obtained from a serum bank that stored residual serum samples from 10 participating hospitals in central and southern Vietnam. The samples were randomly collected from patients regardless of their reason for a hospital visit. In this study, we tested 991 samples, collected from 2013 to 2017; 531 from Ho Chi Minh City (HCMC, southern Vietnam) and 460 from Khanh Hoa (KH, central Vietnam), from age groups 1–30 years old (summarised by year and age group in Table 1). We chose samples up to the age 30 because the majority of individuals have been infected twice before this age in our setting.

**Table 1:** Counts of samples tested for flavivirus antibodies, categorized by age group, year, and location.

	HC (n = 531)					KH (n = 460)				
Age Group	2013	2014	2015	2016	2017	2013	2014	2015	2016	2017
**(0–5]**	31	19	7	53	12	25	36	44	7	0
**(5–10]**	17	10	19	16	20	14	9	6	23	11
**(10–15]**	14	10	10	10	16	02	4	8	26	25
**(15–20]**	17	10	12	8	19	08	5	19	14	15
**(20–25]**	18	17	14	33	10	14	19	9	11	6
**(25–30]**	19	20	20	27	23	15	19	15	20	31
**Total**	116	86	82	147	100	78	92	101	101	88

#### Serological PMA assay

The serological data used in this study were processed by a novel multiplex protein microarray (PMA) developed by Cleton et al. [21]⁠. The PMA slides were printed at the Viroscience Department (Erasmus Medical Centre) as described elsewhere [29]⁠. The slides include several flavivirus antigens such as DENVs 1–4, Zika virus (ZIKV), West Nile virus (WNV), and St. Louis encephalitis virus (SLEV). Selected serum samples were tested for IgG antibodies against these viruses using the protocol previously described [25]^⁠^. Each serum sample was tested serially in 4-fold dilutions, from 1:20 to 1:1280. The antibody concentration indicator is the fluorescent signal with 3,000 and 65,535 as the lower and upper limits of detection respectively. A logistic curve with log-transformation of the signal at 4-dilutions was used to calculate a single PMA titer for each antigen see [25]. From the previous calibration with negative controls, a sample with any titer value against DENV1, DENV2, DENV3, or DENV4 that is higher than 5 is considered positive dengue, otherwise the sample is considered negative.

### Statistical analysis

#### Application of post-infection models to population samples

Statistical models using the PMA assay to infer immune status and past infecting serotype were previously developed and validated on cases with known past infection history by the team [25]⁠. Using the PMA titer profiles against DENV1-4 and other flaviviruses, algorithms were developed that were able to discriminate among different immune statuses—namely, negative, primary, or post-primary infections (Model C). Subsequently, the infected serotype (DENV1-4) of those who had primary infection will be determined using model D. Refer to Thao et al [25] for full detail of the model and data used to build these models.

With the positive dengue samples this current study, we first applied the best predictive model from Thao et al. [25] (model C) to classify dengue immune status (primary or post-primary) for each sample. For the samples classified as primary infections we then applied model D from [25] to infer the infecting serotype of primary infections. In this previous work we could not build a model sufficiently accurate to determine the infecting serotype of post-primary infections, so we did not estimate any serotype for post-primary infections in this current work. After applying these models, we have a dataset CD of DENV seroprevalence of first and second infection with a serotype-specific infection for primary infections that was used for the force of infection estimation in the next step.

Although model D’s accuracy was up to 92% [25], we observed that some samples (12/459) with no cross-reactivity (monotypic reaction) were misclassified. For instance, a sample showed its titer higher than 5 against only DENV1 but was inferred as DENV4. This is an obvious misclassification, so we built a second model in which we first classified those with a response to only one serotype as having been infected with this serotype and then applying model D (now named model F). We thus have a second dataset CF. Eventually, we have two parallel serological datasets to assess how different the result would be if we use these two datasets to estimate the dengue transmission intensity.

#### Force of infection estimation

We applied the multi-serotype catalytic model developed by Ferguson et al. [30] to estimate the FOI of dengue in two locations in Vietnam to our seroprevalence dataset estimated in the previous section. Under the assumption that no cross-reactivity or antibody-dependent enhancement (ADE) response altered risk of infection following a primary infection, the force of infection λ_i_ (a,t) for each dengue serotype was calculated based on the proportion of individuals who were never infected with any serotype, x(a,t), and those who have been exposed solely to serotype i, z_i_(a,t), and those that have been infected more than once [30].


$$\frac{\partial x}{\partial a}+\frac{\partial x}{\partial t}=-x\lambda (a,t-a)$$ (1)

$$\frac{\partial z_{i}}{\partial a}+\frac{\partial z_{i}}{\partial t}=x\lambda (a,t-a)-\sum_{k\neq i}\lambda_{k}(a,t)$$ (2)

The solutions for Eqs (1) and (2) are:

$$x(a,t)=e^{-\int \sum \lambda_{k}(a-\tau ,t-\tau )d\tau }$$ (3)

$$\begin{array}{c} z_{i}(a,t)=[e^{-\int \sum \lambda_{k}(a-\tau ,t-\tau )d\tau }][1-e^{-\int \lambda_{i}(a-\tau ,t-\tau )d\tau }],\\ =x(a,t)[e^{-\int \lambda_{i}(a-\tau ,t-\tau )d\tau }-1] \end{array}$$ (4)

$$=x(a,t)[e^{-\int \lambda_{i}(a-\tau ,t-\tau )d\tau }-1]$$ (5)

Eq (3) represents the probability of escaping infection with any serotype up to time t, whereas Eqs (4–5) indicate the probability of being infected with only serotype i. Therefore, the proportion who have been infected more than once is:

$$1-x(a,t)-\sum z_{i}(a,t).$$ (6)

To better understand the transmission dynamics and intensity of the disease, we explored different assumptions about how the FOI of dengue varies over time, and between serotypes in four models: constant over time and non-serotype specific **(Model 1)**, constant over time and serotype-specific **(Model 2)**, time-varying (annual variation (average for one year), although common to all age groups) and non-serotype specific **(Model 3)**, and time-varying (annual variation, although common to all age groups) and serotype-specific **(Model 4)**.

#### Model fitting

The models were fit to the data using the RStan package [31]⁠. The force of infection parameters of each model were estimated through a Bayesian framework and Markov chain Monte Carlo (MCMC) algorithm [32]. We ran 4 chains, each of 6000 iterations and evaluated convergence by monitoring scale reduction factors, trace plots. We defined a multinomial log-likelihood for parameter inference and assumed the conjugate beta prior distribution, beta(α,β) with parameters α = 2 and β = 5 for the estimation of the force of infection (λ).

Models were compared using the corrected Akaike’s Information Criterion (AICc) and the Bayesian Information Criterion (BIC) [33].

## Results

### Application of post-infection models to population samples

The serum samples were categorized as negative, past primary infection of a particular serotype, or past post-primary infection (Table 2). 38.7% of samples were primary infections, followed by negative (35.8%) and post-primary infections (25.5%). Among primary infections, 47.5% are DENV1, followed by DENV2 (30.0%) and then with many fewer DENV3 (8.1%) and DENV4 (14.4%) infections. There was some variation based on the classification model used, in particular, the number of DENV3 classifications in model F was twice as high as in model D. The dengue serotype-specific prevalence among the two datasets also varies across years (Fig A in S1 Appendix and S1 Table).

**Table 2:** Classification of serum samples. The difference in dengue serotype-specific classifications inferred using model D and model F.

		Model F						
Model D		Negative	Primary				Post-primary	Total
			DENV1	DENV2	DENV3	DENV4		
Negative		355	0	0	0	0	0	355
Primary	DENV1	0	157	11	14	0	0	182
	DENV2	0	11	85	19	0	0	115
	DENV3	0	0	0	31	0	0	31
	DENV4	0	0	10	3	42	0	55
Post-primary		0	0	0	0	0	253	253
Total		355	168	106	67	42	253	991

### Seroprevalence of dengue in HCM and KH

**Fig 1:**
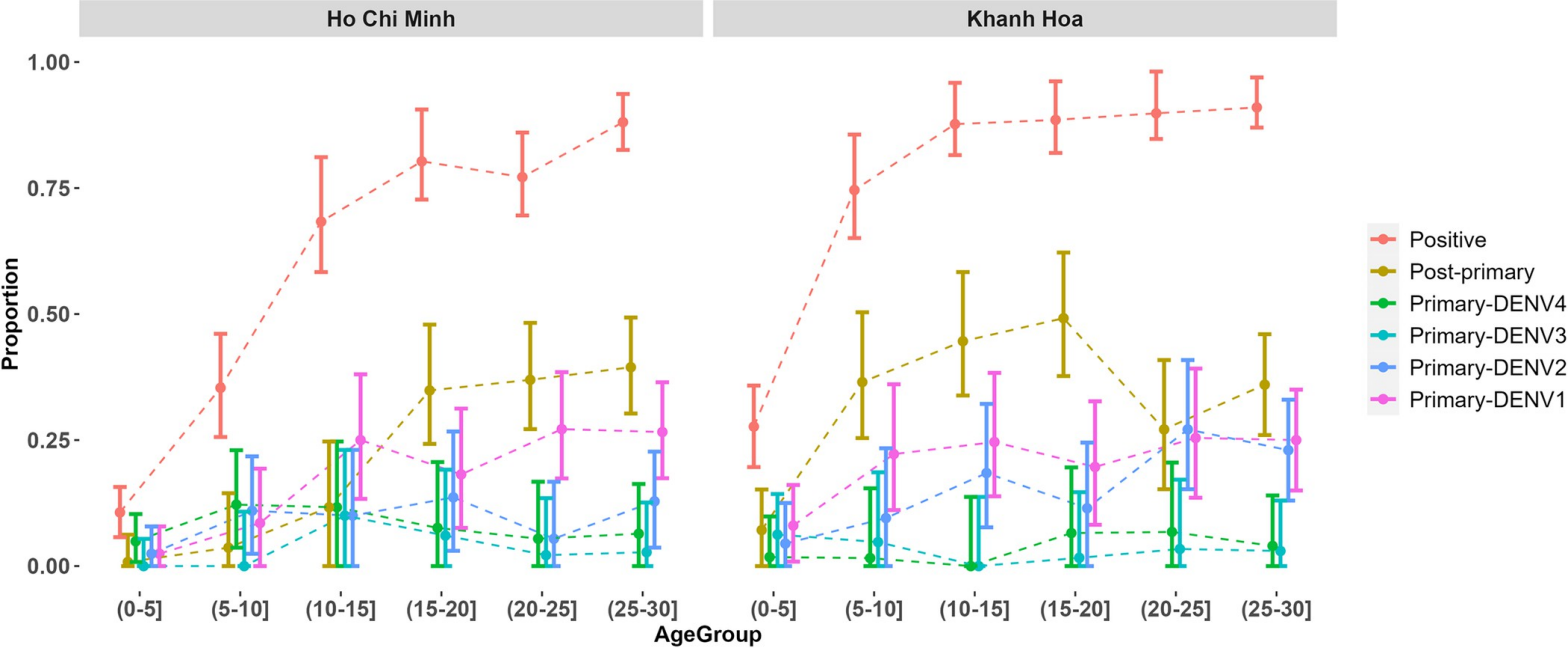
Age-specific and serotype-specific dengue seroprevalence in two locations in Vietnam (2013–2017). The serotype-specific immune status distribution seen are with 95% confidence interval. Positive includes all those with primary and post-primary immune statuses (non-negative).

The overall seroprevalence was estimated to be higher in Khanh Hoa than in Ho Chi Minh, although the pattern of seroprevalence increasing with age was observed in both sites (Figs 1 and S1.). In Ho Chi Minh City, the seroprevalence estimates ranged from 10.7% (95%CI: 5.6–15.7) in the 0-5-year-old group to 88.1% (95%CI: 82.6–93.7) in the 25-30-year-old group. The seroprevalence in Khanh Hoa for the 0-5-year-old group was 27.7% (95%CI: 19.6–35.8) and 91.0% (95%CI, 87.0–97.0) for the 25–30-year-old group.

**Fig 2:**
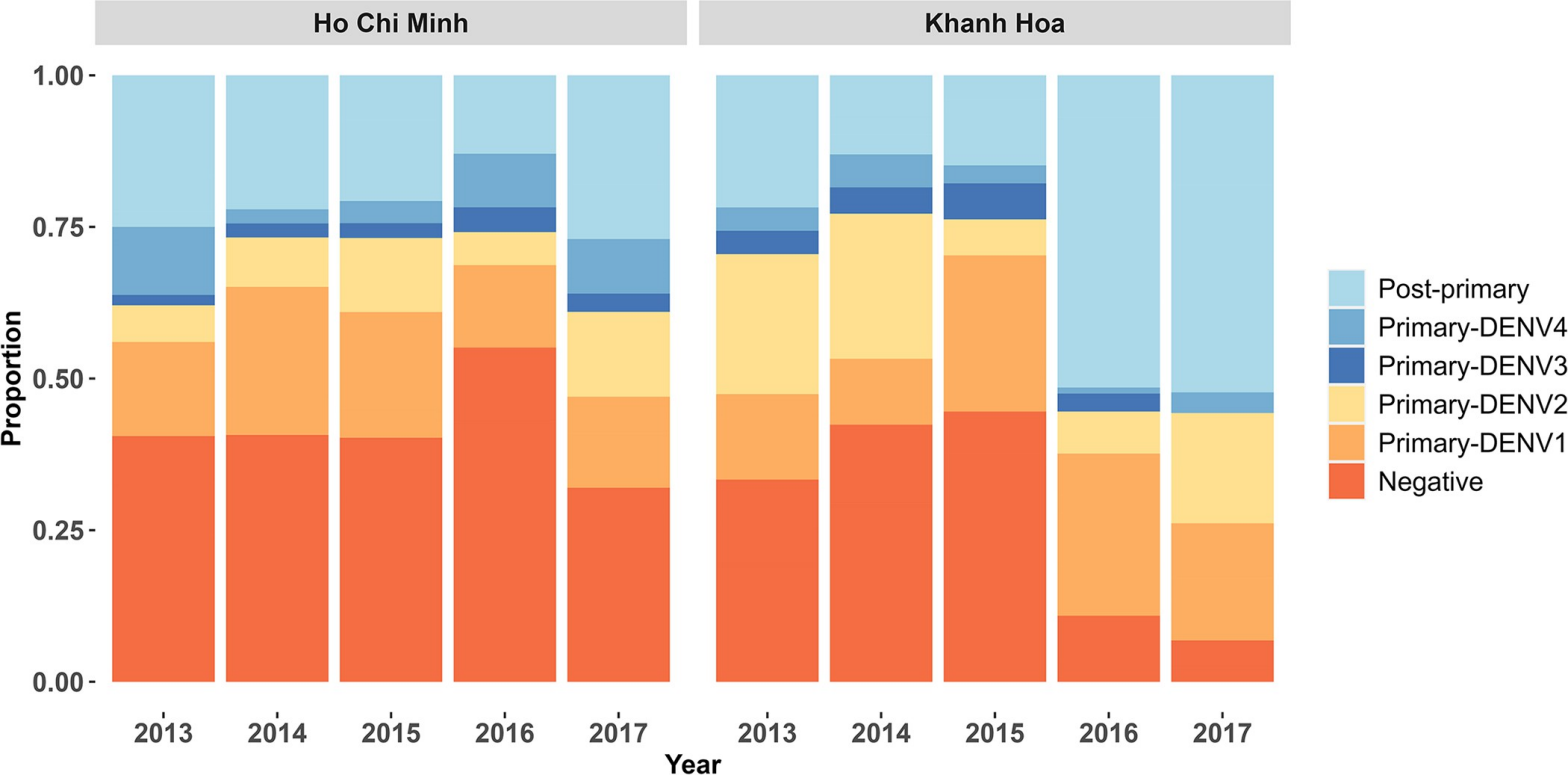
Dengue seroprevalence with immune status predicted by Model C. Immune status was classified as negative, primary and post-primary. Individuals who had antibodies to DENV include primary and post-primary infections. The year is the year of sample collection. The serotype of those primary infections was further inferred by model D.

The proportion of seropositive individuals varies yearly from 2013 to 2017, ranging from 44.9% (95%CI: 36.9–52.4) to 93.2% (95%CI: 82.8–100) (Fig 2 and S1 Table). Past DENV1 was the most common primary infection in Ho Chi Minh City, ranging from 13.6% (95%CI: 6.1–21.7) to 24.4% (95%CI: 1.4–35.6) and remained consistent over the years. In contrast, the predominant past primary infecting serotype in Khanh Hoa switched from DENV2, with 23.1% (95%CI: 12.8–35.2) in 2013 and 23.9% (95%CI:14.1–34.6) in 2014 to DENV1, with 25.7% (95%CI: 16.8–36.5) in 2015 and 26.7% (95%CI: 17.8–37.3) in 2016. In 2017, the proportion was quite similar between the two serotypes, with 19.3% (95%CI: 9.1–29.7) for primary DENV1 and 18.2% (95%CI: 8.0–28.65) for primary DENV2. The switch in predominant serotype happened in 2015 in Khanh Hoa, and there was a surge in post-primary exposures in the next years, with 51.5% (95%CI: 42.6–62.1) in 2016 and 52.3% (95%CI, 42.0–62.7) in 2017.

### Estimating of FOI

**Fig 3:**
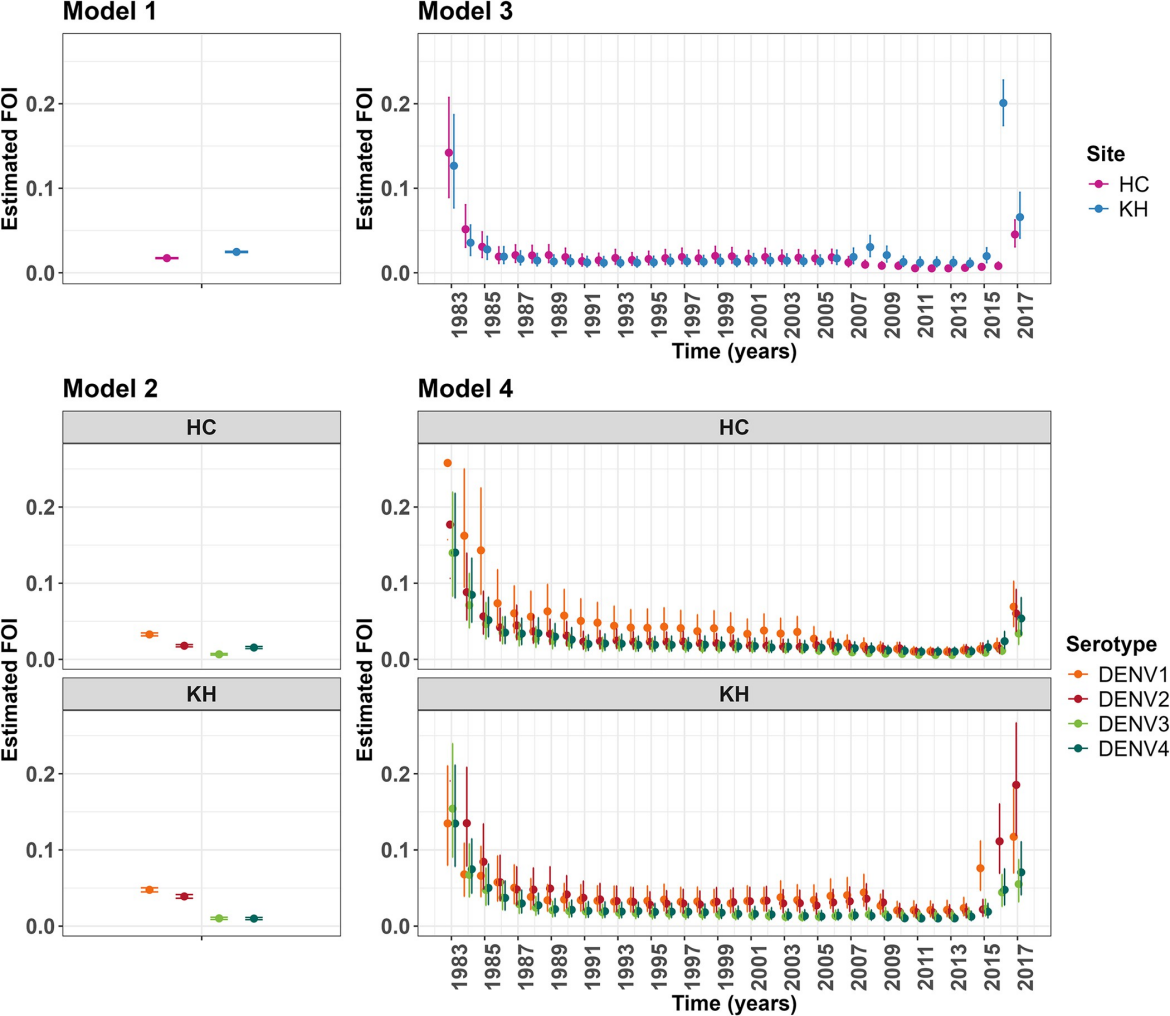
Annual FOI estimates. Results by location with 95% credible interval from four assumptions: constant over time and non-serotype specific **(Model 1)**, constant over time and serotype-specific **(Model 2)**, time-varying (vary yearly but is common to all age groups) and non-serotype specific **(Model 3)**, and time-varying (vary yearly but is common to all age groups) and serotype-specific **(Model 4)**.

For models assuming FOI as constant over time, estimates of the FOI were higher in Khanh Hoa (0.025; 95%CI: 0.024–0.025) than in Ho Chi Minh (0.017; 95%CI: 0.017–0.018) (model 1, Fig 3). This implies that, on average, a higher percentage of the population in Khanh Hoa, by 8%, is exposed to the Dengue virus (DENV) annually compared to Ho Chi Minh. For the serotype specific model (model 2, Fig 3, best fit according to the Bayesian Information Criterion (BIC)), the estimated FOI was highest for DENV1 followed by DENV2 in both locations. While the risk of infection by DENV4 was estimated to be twice as high as DENV3 in Ho Chi Minh, individuals in Khanh Hoa experienced the same risk for these two serotypes, indicating geographical differences in serotype-specific transmission risk. For example, in Ho Chi Minh, the FOI for DENV1 was significantly higher than other serotypes up until 2005.

For models allowing FOI to vary over time (model 3, Fig 3) the results suggest an overall slightly fluctuating trend in FOI estimates from 1986 to 2006 in the two locations, but with somewhat higher values in Ho Chi Minh than in Khanh Hoa. However, since 2007, the risk of dengue infections was decreased in Ho Chi Minh while in Khanh Hoa the risk of infection kept significantly rising until 2009, before it decreased until 2016. A nearly doubling of FOI from 2007 to 2008 in Khanh Hoa from 0.019 (95%CI: 0.011–0.029) to 0.030 (95%CI: 0.019–0.044) marked this reversed trend of a lower risk of dengue infection in Ho Chi Minh City than in Khanh Hoa. In 2016, an abnormal trend was observed in Khanh Hoa when the risk of dengue infection was ten times higher than the previous year, 0.201 (95%CI: 0.174–0.228) compared to 0.020 (95%CI: 0.012–0.029). A similar trend was observed in Ho Chi Minh one year later (2017).

With the model assuming varied transmission yearly and across serotypes, different patterns were observed at each location (model 4, Fig 3). For example, in Ho Chi Minh, the FOI for DENV1 was significantly higher than other serotypes up until 2005. However, from 2006 to 2015, DENV1 transmission intensity was reduced to slightly higher or nearly the same as other serotypes. Noticeably, in 2016, the FOI for DENV4 was highest among the other serotypes. In contrast, in Khanh Hoa the transmission intensity of DENV1 and DENV2 was nearly equal and much higher than that of DENV3 and DENV4 up until 2009. From 2010 to 2014, the risk of infection of DENV1 or DENV2 was still higher than DENV3 or DENV4, but not much different. From 2015 onward, a huge increase in dengue transmission intensity was observed in Khanh Hoa. For example, the FOI for DENV1 in 2015 (0.076; 95%CI: 0.047–0.112) was three times higher than in 2014 (0.024; 95%CI:0.013–0.038). This figure continued increasing in 2016, to 0.364 (95%CI: 0.289–0.441) before dropping down to 0.117 (95%CI: 0.079–0.181) in 2017.

### Assessment of model fit

**Fig 4:**
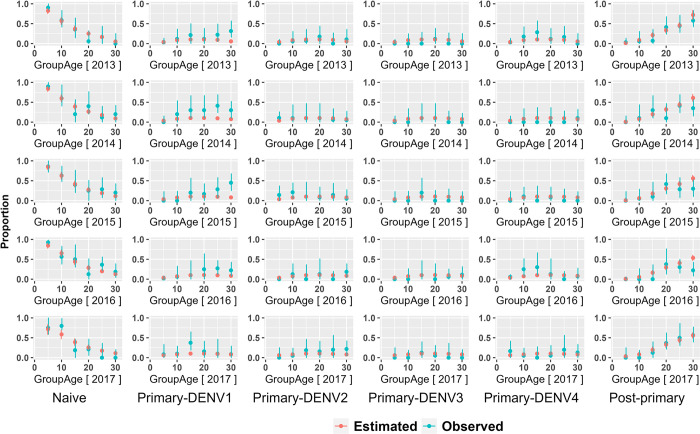
Observed data compared to estimated data from model 3 (the best model according to the Akaike information criterion (AICc)). The Y-axis presents the proportion of dengue immune status regarding naive (left), primary infection with serotype-specific (middle) and post-primary infection (right) of the population in Ho Chi Minh from 2013 to 2017.

We simultaneously used the corrected Akaike information criterion (AICc) and the Bayesian information criterion (BIC) for assessing model fit. Table 3 indicates that the best fit models are model 2 (serotype specific) and model 3 (time varying) using BIC and AICc methods, respectively, regardless of the uncertainty of dataset (CD or CF) and sites (HC or KH). The model fit shown in Fig 4 shows that for all age groups and immune history the model estimates are close to the data.

**Table 3:** Summary of goodness-of-fit. The corrected Akaike information criterion **(**AICc), and the Bayesian information criterion (BIC) of four model assumptions for the two datasets. The model descriptions are as follows. Cons: constant FOI, SS: serotype specific FOI, TV: time varying, TVSS: time varying and serotype specific.

Dataset &Site	Model	Number of estimated parameters (k)	Loglikelihood (LL)	AICc	BIC
CD Ho Chi Minh	Cons (1)	1	-257.28	516.70	517.95
	SS (2)	4	-224.17	457.94	**461.95**
	TV (3)	35	-262.15	**174.30**	643.34
	TVSS (4)	140	-293.71	511.74	1063.59
CF Ho Chi Minh	Cons (1)	1	-244.13	490.40	491.66
	SS (2)	4	-224.16	457.92	**461.93**
	TV (3)	35	-249.10	**148.19**	617.23
	TVSS (4)	140	-290.23	504.78	1056.62
CD Khanh Hoa	Cons (1)	1	-334.74	671.63	672.89
	SS (2)	4	-280.31	570.22	**574.22**
	TV (3)	35	-285.86	**221.73**	690.77
	TVSS (4)	140	-310.37	545.00	1096.84
CF Khanh Hoa	Cons (1)	1	-320.17	642.48	643.74
	SS (2)	4	-277.36	564.32	**568.32**
	TV (3)	35	-271.25	**192.50**	661.54
	TVSS (4)	140	-310.32	544.96	1096.81

## Discussion

We estimated the transmission intensity of dengue (force of infection) over time and by serotype in two regions of Vietnam using the seroprevalence data inferred from a novel flavivirus microarray validated by Thao et al. (25). We estimate a high FOI in HCMC and Khanh Hoa, with estimates of 2–10% of the population getting infected each year, but with substantial variation across years.

It is informative to compare our estimates of force of infection over time and by serotype, for the two areas, to data on (serotype-specific) cases over time. We would not necessarily expect the two to coincide exactly, as the immunity in the population and the serotype circulating would be expected to alter the proportion of infections that result in symptoms and are therefore reported. However, when comparing to the few papers containing information on serotype-specific cases in Vietnam [34–36], we do see that DENV1 is generally the dominant serotype, followed by DENV2, consistent with our force of infection estimates. In Khanh Hoa, we estimated two large peaks in transmission intensity in 2008 and 2015, with higher transmission from 2015 onwards, with a sharp increase of the transmission intensity in Khanh Hoa from 0.020 (95%CI: 0.012–0.029) in 2015 to 0.201 (95%CI: 0.174–0.228) in 2016. The 2015 peak is consistent with the case notifications in Nha Trang (the capital city of Khanh Hoa), as reported by Quyen et al. [34]. Such an increase in dengue transmission intensity could be attributed to the cessation of the National Dengue Control Program (NDCP) in Vietnam since 2015 [37]. Indeed, the national surveillance data showed that the average annual dengue incidences were 7,722 and 2,968 cases in Ho Chi Minh and Khanh Hoa, respectively, from 1998 to 2015. Since 2015, these figures increased to nearly triple in Ho Chi Minh (21,491 cases/year) and double in Khanh Hoa (5,782 cases/year). (surveillance’s data from S2 Fig).

In addition, the dominance of primary DENV1 in 2014 was observed to be switched to DENV2 in 2015 in Khanh Hoa. This shift in serotype dominance may have contributed to the high secondary rate observed in 2016, with a lag of one year from the serotype switch. However, it is important to note that the prevalence in a certain year (e.g., 2016) does not necessarily indicate that infections occurred solely in that year; they could have occurred in any years leading up to that specific year (e.g., up to 2016). Thus, the observed increase in secondary exposures in 2016 could reflect infections from previous years. Another possible explanation for this delay could be the time required for the new serotype to become prevalent in the population, leading to a higher number of secondary exposures. This lag in the increase of secondary exposures following a predominant serotype switch highlights the complex dynamics of dengue virus transmission and warrants further investigation.

There are some specific questions raised by the Khanh Hoa data and transmission patterns. The high recent transmission means that it is necessary to analyse the data, as we have, by year and grouping the data can lead to masking of the true transmission. In addition, due to the possibility of cross-reactivity with Zika, and uncertainty about Zika transmission in Vietnam during the 2015–2016 outbreak, we tested to see if the observed patterns in Khanh Hoa could be explained by past ZIKV transmission. Analysis of ZIKV from the PMA showed that as well as the increase in dengue titres, there were higher titres against ZIKV from 2016 as compared to previous years (S3 Fig). This could be linked to the Zika virus outbreak from 2015 to 2016 [38,39] or, as we are currently assuming, be due to cross-reactivity with DENVs that were circulating that year. Hence, whether the estimated large peaks in transmission intensity in 2015 can be attributed to increased DENV transmission or cross-reactivity to immune response from ZIKV infection remains uncertain, warranting further investigation. The further investigation could include the assessment of the assay on follow up from Zika samples as was previously undertaken for dengue by Thao et al. [25].

We have shown that the use of PMA assay, combined with extended catalytic models of multiple exposures of dengue can increase what can be estimated about transmission intensity for serological studies. In applying the validated PMA assay to the population, the pattern we inferred with age i.e. people with older age are more likely to have had a second infection (Fig 1) is consistent with the epidemiological patterns of age in Vietnam [34,40]. This is further evidence that the assessment of number of past infections (zero, once or more than once) from the PMA assay is reliable. Moreover, this method allows us to discriminate which dengue serotypes an individual has been infected with after their first infection, thus providing the ability to estimate serotype specific FOI and to estimate the FOI in more detail from cross-sectional studies. Our estimates of the DENV FOI were comparable across a variety of assumptions. However, although the goodness-of-fit suggested that models with time-varying or serotype-specific FOI resulted in the best fit to the data (Table 3), whether the variation was serotype-specific or time still remains difficult to determine. However still, our results provide more detailed information on serotype specific virus transmission dynamics due to the additional information of serological data generated from the PMA assay.

There are a number of limitations to the analysis. The samples used are from a convenience sample not a random sample of the population. However, convenience sampling is widely used as it is a way to collect more samples, cheaply [41,42]. In previous work where it was possible to compare we found our dengue seroprevalence estimates consistent with randomly collected samples [27]. We have limited samples for some ages and years and therefore greater uncertainty on some of the estimates. In addition, we had no information on what the response in this array is after infection with other flaviviruses, in particular Zika. Moreover, we did not consider age-varying FOI in our models, assuming that FOI was homogenous across all age groups for a given year or DENV serotype. Furthermore, only using seroprevalence data from recent years (2013–2017) might lead to a potential recency bias in estimating the force of infection (FOI). As seen in Fig 3 (models 3 and 4), the FOI values at the right end of the graph correspond to samples collected in the same year as the FOI estimation. At the same time, the FOI estimates from the 1980s and 1990s correspond to individuals who were infected 25 years ago and might experience antibody waning over time. This raises the possibility that the FOI estimates may be skewed towards current infection rates, while long-term trends may not be fully captured. We have made the assumption that during any cross-protected period, the risk of seroconverting does not change, which, though consistent with estimates from cohorts [15], could be further explored in future model iterations. With serological data however, we are not making any assumption about the risk of symptomatic or severe disease in this period. Lastly, our models were fitted to samples from Vietnam, a dengue-endemic country. Whether our models are able to perform in lower transmission settings warrants further investigation and will require a wider age range of samples.

The next steps for this work will be combined estimation using case data (serotype-specific where available) and seroprevalence data. A greater amount of serotype specific case data would be valuable here. Furthermore, we plan to incorporate this serological data (particularly the proportion susceptible to first and second infections of the different serotypes) into prediction models to assess whether it improves prediction. Serotype specific understanding of past transmission and population immunity profiles (including numbers of past infections) is of particular current importance due to the profiles of current available vaccines, including variable serotype efficacy and differential performance depending on past infection history [43,44]. We have shown here variation in transmission between two locations in Vietnam, and information from further locations would be useful for rationale vaccination use in different locations.

## Conclusion

We present analysis updating the estimates of the serotype specific transmission intensity in two regions in Vietnam over time. The use of the PMA on serial age-stratified cross-sectional samples increases the amount of information available, and should be considered for future serological surveys for dengue. However, there are still limitations to what can be inferred due to the remaining cross-reactivity. These estimates are useful for understanding past transmission, the impact of control measures and for planning of future control measures, including vaccination and vector control.

## Supporting information

S1 Appendix Dataset CD and CF. (DOCX)

S1 Fig Estimated dengue seroprevalence by age. (DOCX)

S2 Fig Surveillance data. (DOCX)

S3 Fig PMA titres against Dengue serotypes 1–4 and Zika virus for each individual. (DOCX)

S1 Table Yearly serotype-specific prevalence, 2013–2017. (DOCX)
