# Supplementary material for: Estimating the force of infection of four dengue serotypes from serological studies in two regions of Vietnam
Source: PLoS Negl Trop Dis. 2024 Oct 7;18(10):e0012568. doi: 10.1371/journal.pntd.0012568 (PMC11521262; doi:10.1371/journal.pntd.0012568)
Supplement: S1 Appendix — (DOCX) [file pntd.0012568.s001.docx]

**S1 Appendix. Dataset CD and CF**

| **Model** | **Outcome** | **Predictors** | **Accuracy** |
| --- | --- | --- | --- |
| C ^(a)^ | Primary or secondary infection | DENV3 + DENV4 + ZIKV + SLEV + WNV | 0.85 |
| D ^(a)^ | Infecting DENV serotype | DENV1 + DENV2 + DENV3 + DENV4 | 0.92 |
| F | Firstly, the serotype is assigned for the homotypic sample (b), and then model D is applied. | | |

(a) Models were developed by Thao et al,. (1)

(b) Each sample has titer values against different flaviviruses spotted on the PMA slide. The homotypic sample is defined as those with only one titer higher than the cutoff titer to be considered a positive sample. Therefore, the virus with this high-value titer must be the infecting serotype.

In summary, both datasets initially infer immune status (primary or post-primary infection) using model C. Subsequently, primary cases were further classified into different serotypes using model D, resulting in dataset CD. On the other hand, for dataset CF, primary cases were first identified, and homotypic serotypes were manually assigned before applying model D.

**
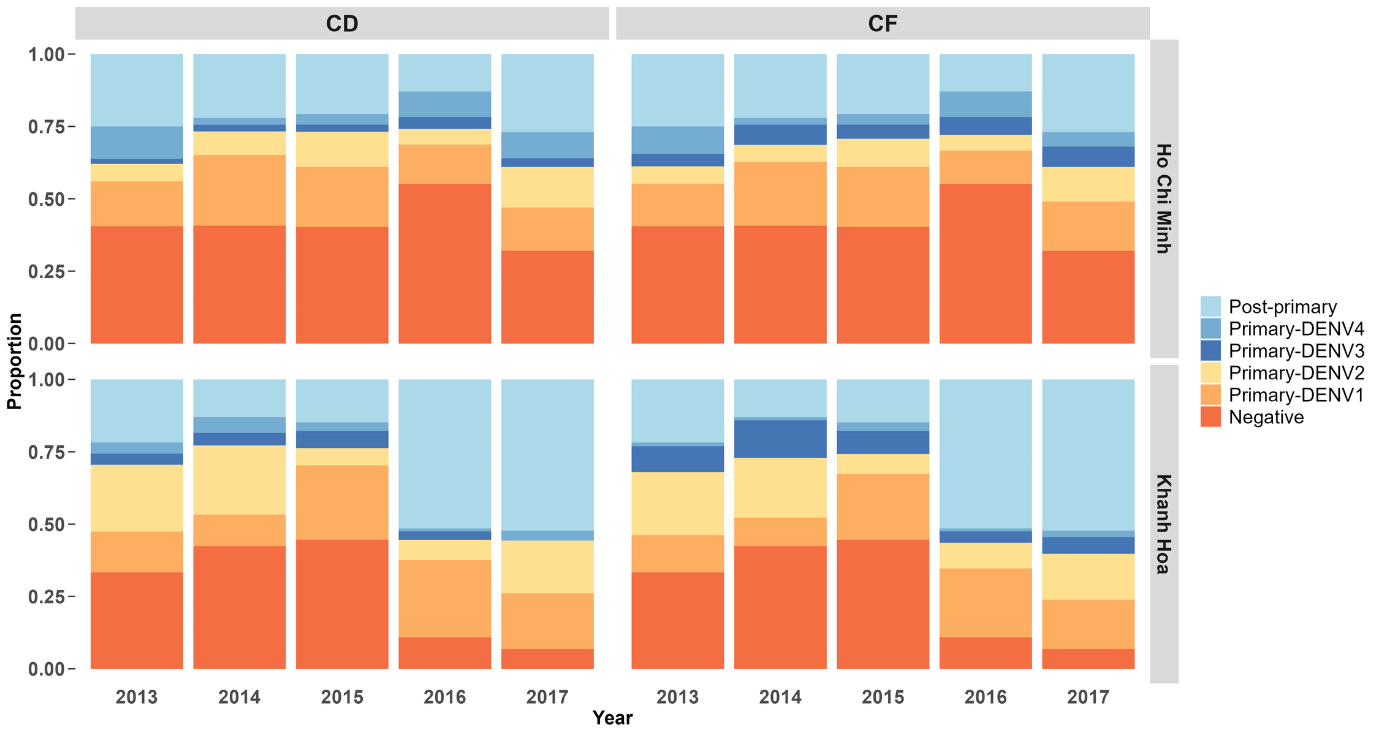
**

**Fig A. Dengue seroprevalence with immune status predicted by model C.** Immune status negative, primary and secondary were used. The serotype-specific of those primary infections was further inferred by model D or model F.

**Reference**

1. Thao TTN, de Bruin E, Phuong HT, Thao Vy NH, van den Ham HJ, Wills BA, et al. Using NS1 Flavivirus Protein Microarray to Infer Past Infecting Dengue Virus Serotype and Number of Past Dengue Virus Infections in Vietnamese Individuals. J Infect Dis. 2021 Jun 15;223(12):2053–61.
