# Supplementary material for: Estimating the force of infection of four dengue serotypes from serological studies in two regions of Vietnam
Source: PLoS Negl Trop Dis. 2024 Oct 7;18(10):e0012568. doi: 10.1371/journal.pntd.0012568 (PMC11521262; doi:10.1371/journal.pntd.0012568)
Supplement: S1 Fig — (DOCX) [file pntd.0012568.s002.docx]

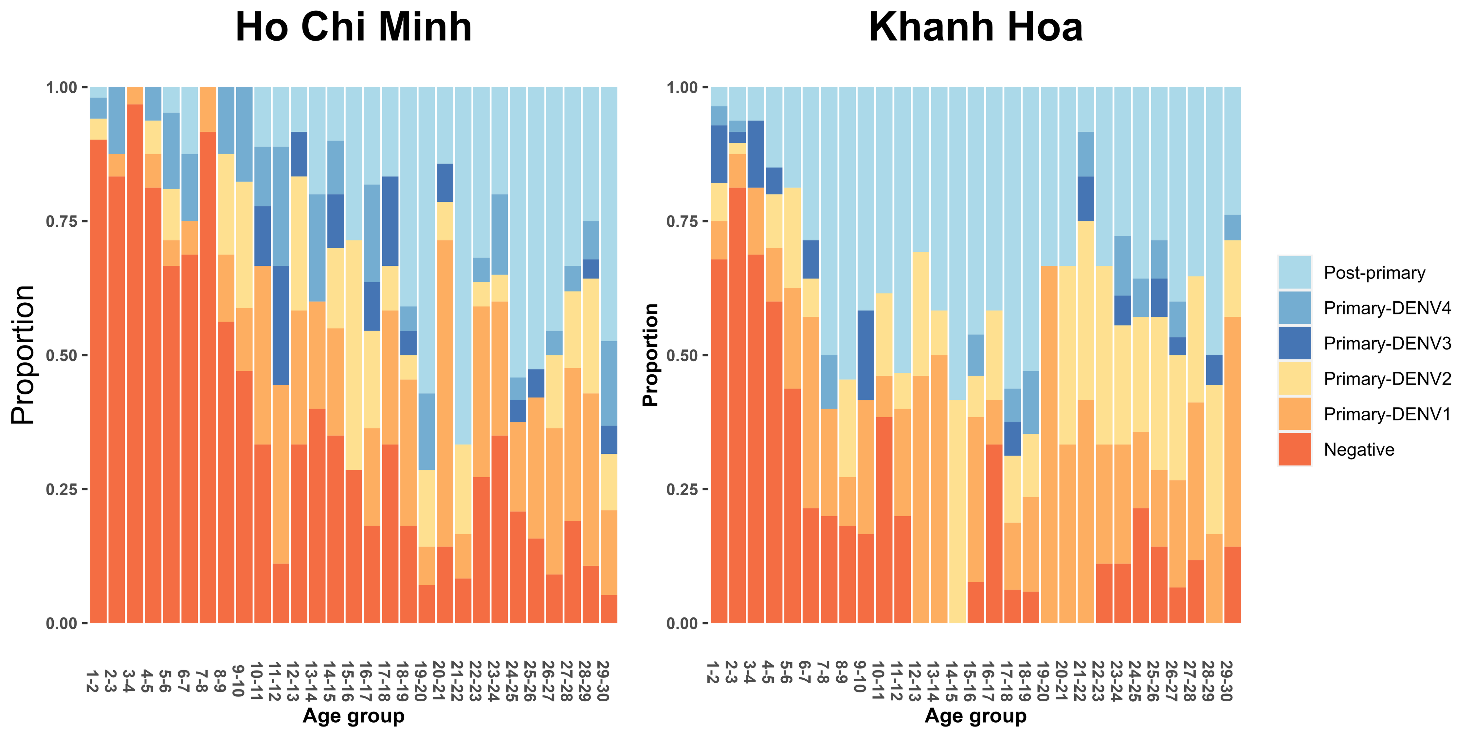


**S1 Fig.** **Estimated dengue seroprevalence by age**. Proportion of negative (naive), primary infections by serotype, and secondary infections were estimated. Data were collected from individuals between one and thirty years of age in Ho Chi Minh (n= 531) and Khanh Hoa (n=460), Vietnam, between 2013-2017.
