# Supplementary material for: Estimating the force of infection of four dengue serotypes from serological studies in two regions of Vietnam
Source: PLoS Negl Trop Dis. 2024 Oct 7;18(10):e0012568. doi: 10.1371/journal.pntd.0012568 (PMC11521262; doi:10.1371/journal.pntd.0012568)
Supplement: S2 Fig — (DOCX) [file pntd.0012568.s003.docx]

**
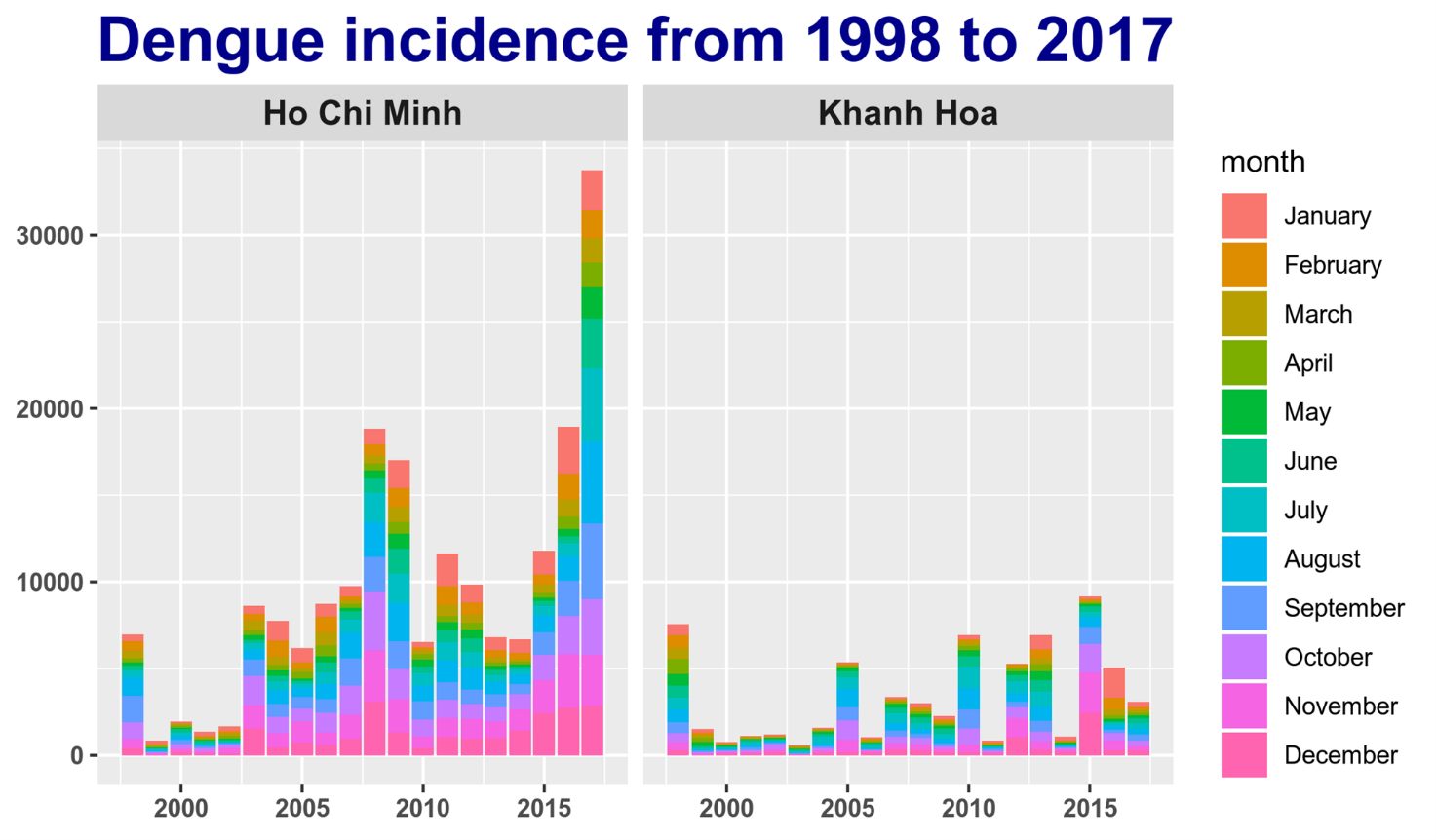
**

**S2 Fig. Surveillance data.** Dengue case notifications as part of Vietnam's national dengue control program, 1998-2017 (data from National Institute of Hygiene and Epidemiology (NIHE)).
