# Supplementary material for: Estimating the force of infection of four dengue serotypes from serological studies in two regions of Vietnam
Source: PLoS Negl Trop Dis. 2024 Oct 7;18(10):e0012568. doi: 10.1371/journal.pntd.0012568 (PMC11521262; doi:10.1371/journal.pntd.0012568)
Supplement: S3 Fig — (DOCX) [file pntd.0012568.s004.docx]

**
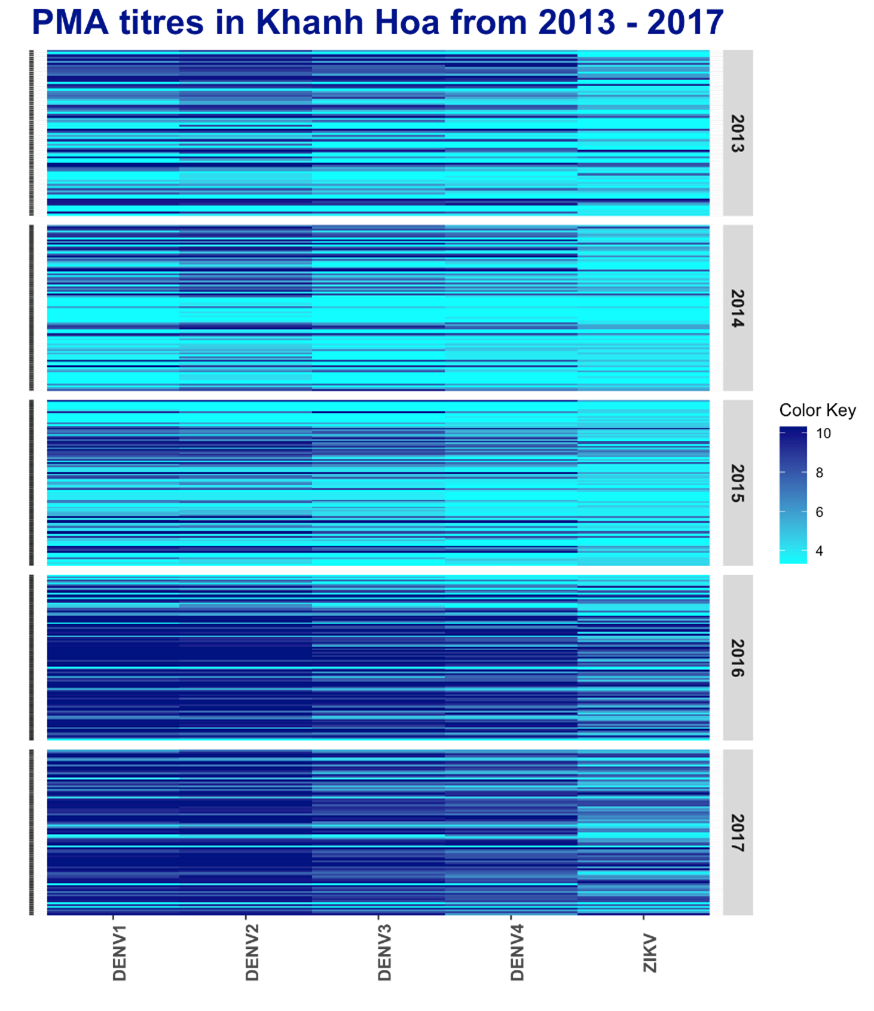
**

**S3 Fig.** **PMA titres against Dengue serotypes 1-4 and Zika virus for each individual.** Data were collected from 460 individuals residing in Khanh Hoa, between 2013 and 2017, with each row corresponding to a unique individual. Higher titre values are observed across all viruses being tested by the PMA from 2016.
