# Supplementary material for: Estimating the force of infection of four dengue serotypes from serological studies in two regions of Vietnam
Source: PLoS Negl Trop Dis. 2024 Oct 7;18(10):e0012568. doi: 10.1371/journal.pntd.0012568 (PMC11521262; doi:10.1371/journal.pntd.0012568)
Supplement: S1 Table — (DOCX) [file pntd.0012568.s005.docx]

**S1 Table. Yearly serotype-specific prevalence, 2013-2017.** Proportions of negative (naive), primary infections and post-primary infections are shown for each year at different sites.

|  | **Seroprevalence percentage (95%CI)** | | | | |
| --- | --- | --- | --- | --- | --- |
| **Site, dataset** | 2013 | 2014 | 2015 | 2016 | 2017 |
| HC, CD |  |  |  |  |  |
| Naive | 40.5 (31.9-50.4) | 40.7 (30.2-51.9) | 40.2(30.5-52.3) | 55.1 (47.6-63.2) | 32.0 (23.0-42.7) |
| Primary |  |  |  |  |  |
| DENV1 | 15.5 (6.9-25.4) | 24.4 (1.4-35.6) | 20.7 (11-32.7) | 13.6 (6.1-21.7) | 15.0 (6.0-25.7) |
| DENV2 | 6.0 (0.0-15.9) | 8.1 (0.0-19.3) | 12.2 (2.4-24.2) | 5.4 (0.0-13.5) | 14.0 (5.0-24.7) |
| DENV3 | 1.7 (0.0-11.6) | 2.3 (0.0-13.5) | 2.4 (0.0-14.4) | 4.1 (0.0-12.2) | 3.0 (0.0-13.7) |
| DENV4 | 11.2 (2.6-21.1) | 2.3 (0.0-13.5) | 3.7 (0.0-15.7) | 8.8 (1.4-16.9) | 9.0 (0.0-19.7) |
| Post-primary | 25.0 (16.4-34.9) | 22.1 (11.6-33.3) | 20.7 (11.0-32.7) | 12.9 (5.4-21.0) | 27.0 (18.0-37.7) |
| KH, CD |  |  |  |  |  |
| Naive | 33.3 (23.1-45.5) | 42.4 (32.6-53.1) | 44.6 (35.6 -55.3) | 10.9 (2.0-21.5) | 6.8 (0.0-17.2) |
| Primary |  |  |  |  |  |
| DENV1 | 14.1 (3.8-26.2) | 10.9 (1.1-21.6) | 25.7 (16.8-36.5) | 26.7 (17.8-37.3) | 19.3 (9.1-29.7) |
| DENV2 | 23.1 (12.8-35.2) | 23.9 (14.1-34.6) | 5.9 (0.0-16.7) | 6.9 (0.0- 17.5) | 18.2 (8.0-28.6) |
| DENV3 | 3.8 (0.0-16.0) | 4.3 (0.0-15.1) | 5.9 (0.0-16.7) | 3.0 (0.0-13.6) | 0.0 (0.0-10.4) |
| DENV4 | 3.8 (0.0 -16.0) | 5.4 (0.0-16.2) | 3.0 (0.0-13.7) | 1.0 (0.0-11.6) | 3.4 (0.0-13.8) |
| Post-primary | 21.8 (11.5-33.9) | 13.0 (3.3-23.8) | 14.9 (5.9-25.6) | 51.5 (42.6-62.1) | 52.3 (42.0-62.7) |
| HC, CF |  |  |  |  |  |
| Naive | 40.5 (31.9-50.3) | 40.7 (30.2-51.7) | 40.2 (30.5-52.2) | 55.1 (47.6-63.1) | 32.0 (23.0-42.7) |
| Primary |  |  |  |  |  |
| DENV1 | 14.7 (6.0 -24.5) | 22.1 (11.6-33.1) | 20.7 (11.0-32.7) | 11.6 (4.1 -19.6) | 17.0 (8.0-27.7) |
| DENV2 | 6.0 (0.0-15.8) | 5.8 (0.0-16.8) | 9.8 (0.0-21.7) | 5.4 (0.0-13.4) | 12.0 (3.0-22.7) |
| DENV3 | 4.3 (0.0-14.1) | 7.0 (0.0-18.0) | 4.9 (0.0-16.8) | 6.1 (0.0-14.1) | 7.0 (0.0-17.7) |
| DENV4 | 9.5 (0.9-19.3) | 2.3 (0.0-13.3) | 3.7 (0.0.0-15.6) | 8.8 (1.4-16.8) | 5.0 (0.0-15.7) |
| Post-primary | 25.0 (16.4-34.8) | 22.1 (11.6-33.1) | 20.7 (11.0 -32.7) | 12.9 (5.4-20.9) | 27.0 (18.0-37.7) |
| KH, CF |  |  |  |  |  |
| Naive | 33.3 (23.1-45.4) | 42.4 (32.6-53.0) | 44.6 (35.6-55.2) | 10.9 (2.0-21.4) | 6.8 (0.0-18) |
| Primary |  |  |  |  |  |
| DENV1 | 12.8 (2.6-24.8) | 9.8 (0.0-20.4) | 22.8 (13.9-33.4) | 23.8 (14.9-34.2) | 17.0 (8.0-28.3) |
| DENV2 | 21.8 (11.5-33.8) | 20.7 (10.9-31.2) | 6.9 (0.0-17.6) | 8.9 (0.0-19.4) | 15.9 (6.8-27.1) |
| DENV3 | 9.0 (0.0-21.0) | 13.0 (3.3-23.6) | 7.9 (0.0-18.5) | 4.0 (0.0-14.4) | 5.7 (0.0-16.9) |
| DENV4 | 1.3 (0.0-13.3) | 1.1 (0.0-11.7) | 3.0 (0.0-13.6) | 1.0 (0.0-11.5) | 2.3 (0.0-13.5) |
| Post-primary | 21.8 (11.5-33.8) | 13.0 (3.3-23.6) | 14.9 (5.9-25.5) | 51.5 (42.6-62.0) | 52.3 (43.2-63.5) |
